# Construction and Analysis of Competing Endogenous RNA Networks for Breast Cancer Based on TCGA Dataset

**DOI:** 10.1155/2020/4078596

**Published:** 2020-07-24

**Authors:** Xue Wang, Chundi Gao, Fubin Feng, Jing Zhuang, Lijuan Liu, Huayao Li, Cun Liu, Jibiao Wu, Xia Zheng, Xia Ding, Changgang Sun

**Affiliations:** ^1^College of Basic Medicine, Qingdao University, Qingdao, Shandong Province, China 266000; ^2^College of First Clinical Medicine, Shandong University of Traditional Chinese Medicine, Jinan, Shandong Province, China 250000; ^3^Department of Oncology, Weifang Traditional Chinese Hospital, Weifang, Shandong Province, China 261000; ^4^Department of Oncology, Affiliated Hospital of Weifang Medical University, Weifang, Shandong Province, China 261000; ^5^College of Basic Medical, Shandong University of Traditional Chinese Medicine, Jinan, Shandong Province, China 250000; ^6^College of Traditional Chinese Medicine, Shandong University of Traditional Chinese Medicine, Jinan, Shandong Province, China 250000; ^7^School of Traditional Chinese Medicine, Beijing University of Traditional Chinese Medicine, Beijing Province, China 100029; ^8^Cancer and Immunology Institute, Shandong University of Traditional Chinese Medicine, Jinan, Shandong Province, China 250000

## Abstract

**Background:**

Long noncoding RNAs (lncRNAs) act as competing endogenous RNAs for microRNAs in cancer metastasis. However, the roles of lncRNA-mediated competing endogenous RNA (ceRNA) networks for breast cancer (BC) are still unclear. *Material and Methods*. The expression profiles of mRNAs, lncRNAs, and miRNAs with BC were extracted from The Cancer Genome Atlas database. Weighted gene coexpression network analysis was conducted to extract differentially expressed mRNAs (DEmRNAs) that might be core genes. Through miRWalk, TargetScan, and miRDB to predict the target genes, an abnormal lncRNA-miRNA-mRNA ceRNA network with BC was constructed. The survival possibilities of mRNAs, miRNAs, and lncRNAs for patients with BC were determined by Kaplan-Meier survival curves and Oncomine.

**Results:**

We identified 2134 DEmRNAs, 1059 differentially expressed lncRNAs (DElncRNAs), and 86 differentially expressed miRNAs (DEmiRNAs). We then compose a ceRNA network for BC, including 72 DElncRNAs, 8 DEmiRNAs, and 12 DEmRNAs. After verification, 2 lncRNAs (LINC00466, LINC00460), 1 miRNA (Hsa-mir-204), and 5 mRNAs (TGFBR2, CDH2, CHRDL1, FGF2, and CHL1) were meaningful as prognostic biomarkers for BC patients. In the ceRNA network, we found that three axes were present in 10 RNAs related to the prognosis of BC, namely, LINC00466-Hsa-mir-204-TGFBR2, LINC00466-Hsa-mir-204-CDH2, and LINC00466-Hsa-mir-204-CHRDL1.

**Conclusion:**

This study highlighted lncRNA-miRNA-mRNA ceRNA related to the pathogenesis of BC, which might be used for latent diagnostic biomarkers and therapeutic targets for BC.

## 1. Introduction

Breast cancer (BC) is one of the most popular malignant cancers in women. Annually, approximately 1.7 million women worldwide are diagnosed with BC, and its morbidity and mortality rates continue to soar [1]. At present, there are two main treatments for breast cancer, including chemotherapy and surgery [2]. However, due to the complexity of the disease mechanism of BC, the effectiveness of chemotherapy is unsatisfactory and the recurrence rate is high. Therefore, the molecular pathogenesis and identification of BC require an in-depth understanding. This immediately requires discovering new candidate therapeutic targets and biomarkers correlated with the clinical status of BC. Analyzing bioinformatics-based tumor molecular pathogenesis not only explores the tumors' molecular pathogenesis but also identifies new biomarkers of tumorigenesis and prognosis. This is important for early diagnosis of BC, improving its prognosis, and reducing its incidence.

Numerous studies on genome sequencing and transcriptome analysis have validated many RNAs, long noncoding RNAs (lncRNAs), and microRNAs (miRNAs) [3]. lncRNAs are a type of noncoding RNA that contain many biological species. Recent researches have shown that lncRNAs act a pivotal part in the interaction of various organisms, such as chromatin modification, nuclear transport, organogenesis, and tumorigenesis [4]. Competitive endogenous RNA (ceRNA) networks act an important part in posttranscriptional regulation. lncRNA function as ceRNAs by binding miRNA to messenger RNAs (mRNAs). Many lncRNAs bind to miRNAs and serve as miRNA sponges, thus releasing miRNAs from target genes and improving the expression level of target genes [5]. These interactions provide an important opportunity for us to further understand cancer treatment. Based on previous studies, the lncRNA-miRNA-mRNA ceRNA networks have been confirmed. For example, Yang et al. demonstrated that ARNILA is a lncRNA that plays a part in ceRNA for miR-204 to promote the expression of SOX4, which is known to induce epithelial-mesenchymal transition (EMT) and contribute to BC progression, thereby promoting EMT, invasion, and metastasis of triple-negative BC [6]. Chen et al. explained that lncRNA UICLM acted as a ceRNA for miR-215 to facilitate the expression of its target gene ZEB2, offering a new prediction target for colorectal cancer [3]. However, there are few analyses by the synthesis of BC-related lncRNA-miRNA-mRNA ceRNA network in a large sample size.

In this paper, we attempted to discuss the interaction between lncRNA-miRNA-mRNA based on ceRNA theory of BC. We obtained meaningful differentially expressed lncRNAs, miRNAs, and mRNAs from The Cancer Genome Atlas (TCGA) database. Key molecule genes were identified by performing weighted gene coexpression network analysis (WGCNA) and functional annotation. We structured the ceRNA network that contained 72 lncRNAs, 8 miRNAs, and 12 mRNAs. Survival analysis and use of Oncomine were aimed at verifying the ceRNA. Ultimately, 2 lncRNAs (LINC00466, LINC00460), 1 miRNA (Hsa-mir-204), and 5 mRNAs (TGFBR2, CDH2, CHRDL1, FGF2, and CHL1) were meaningful prognostic biomarkers in BC patients. Through this study, we do enhance the understanding of BC's molecular mechanism as well as provide potential diagnostic biomarkers of lncRNA, miRNA, and mRNA for BC, which will be conducive to patients with early diagnosis, treatment, and prognosis.

## 2. Material and Methods

### 2.1. Data Source

The RNA expression profile (112 normal tissues, 1096 tumor tissues), miRNA (44 normal tissues, 525 tumor tissues), and corresponding patient clinical follow-up data were downloaded from TCGA [7]. The lncRNA and mRNA expression profiles were then isolated from the RNA expression profiles.

### 2.2. Identification of DEmRNAs, DElncRNAs, and DEmiRNAs

To distinguish the mRNAs, lncRNAs, and miRNAs that are differentially expressed between the breast cancer with the normal samples, the downloaded mRNA, lncRNA, and miRNA data were standardized and differential expression analysis was conducted applying the edgeR software package of R software suite. Fold change (FC, the mean ratio of the expression of the transcript in cancer samples to that in normal) was used to measure the degree of change. The *P* value was used to determine the saliency threshold of multiple trials. The False Discovery Rate (FDR) is obtained by correcting *P* value of different significance. The setting conditions were FDR < 0.01 and ∣log2FC  | >2.

### 2.3. Correspondence between DEmiRNAs and DElncRNAs

The interrelationship between miRNA and lncRNA was predicted through the miRcode database, which contains 179,905 transcripts, 53,520 genes, and 10,419 lncRNAs. The database allows whole transcriptome human miRNA target predictions according to GENCODE gene annotation.

### 2.4. Network Analysis of the DEmRNAs

We analyzed the merged network by WGCNA. It is a systematic biological method used to describe gene association patterns among different samples, and it can be used for identifying highly collaborative gene sets. The candidate biomarker genes or therapeutic targets were identified according to the linkage of the gene set and the relationship between the gene set and phenotype [8, 9]. To identify the DEmRNAs, we set power criteria of 6 and module size criteria of 15.

### 2.5. Functional Annotation

To explain the interrelationship of the biological mechanisms connected with BC genes that are identified with WGCNA, we used the DAVID database to associate genes with biological annotations at the statistical level, to identify the most significantly enriched biological annotations out of thousands of associated annotations.

### 2.6. Correspondence between DEmRNAs and DEmiRNAs

We used miRWalk, TargetScan, and miRDB to seek DEmiRNA-mRNAs. miRWalk is a fully archived and freely available database that provides the largest set of predictive and experimentally proven interactions between microRNA targets in a variety of novel ways [10]. TargetScan is a software specially designed to analyze target genes of mammalian miRNA, which can play a posttranscriptional regulatory role by binding the 3′UTR region of the transcript sequence [11]. miRDB is an online database that can predict targets and annotate functions for miRNA by analyzing the interaction between miRNA and target in high-throughput sequencing experiment [12].

### 2.7. Setup of the ceRNA Network

**Figure 1:**
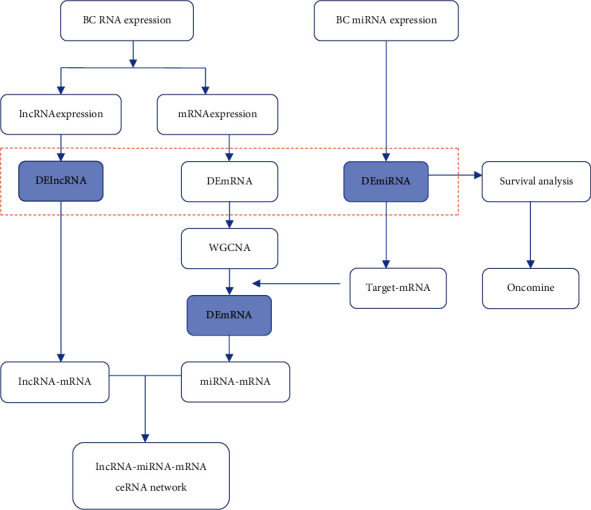
Main procedure followed when constructing the ceRNA network.

To construct the ceRNA network in BC, the miRcode database was applied to appraised miRNA-lncRNA relations. Then, miRWalk, TargetScan, and miRDB were educed miRNA-targeted mRNAs. The results intersect with the core modules of WGCNA. The major process of the article is displayed in Figure 1.

### 2.8. Analysis of Gene Expression Levels

Survival of 2 lncRNA, 1 miRNA, and 5 genes in ceRNA was analyzed. Furthermore, Oncomine (https://http://www.oncomine.org) is a web-based data mining platform containing 715 datasets and 86733 samples [13]. Oncomine has a systematic cancer mutation spectrum, gene expression data, and relevant clinical information, which can be used to find new biomarkers or new therapeutic targets. Oncomine was used to analyze the mRNA level in BC tissue. A *P* value < 1*E*-4 and FC > 2 act as standard conditions.

## 3. Results

### 3.1. lncRNAs, miRNAs, and mRNAs of Differential Expression in BC Patients

The expression profiles of patients with BC from the TCGA database were obtained. After differential expression analysis, 1059 lncRNAs, 86 miRNAs, and 2134 mRNAs were identified as being abnormal expression with FDR < 0.01 and ∣log2FC | >2. Of these, 1375 mRNAs, 842 lncRNAs, and 67 miRNAs were upregulated and 759 mRNAs, 217 lncRNAs, and 19 miRNAs were downregulated in BC patients.

### 3.2. Weighted Gene Coexpression Network Analysis

**Figure 2:**
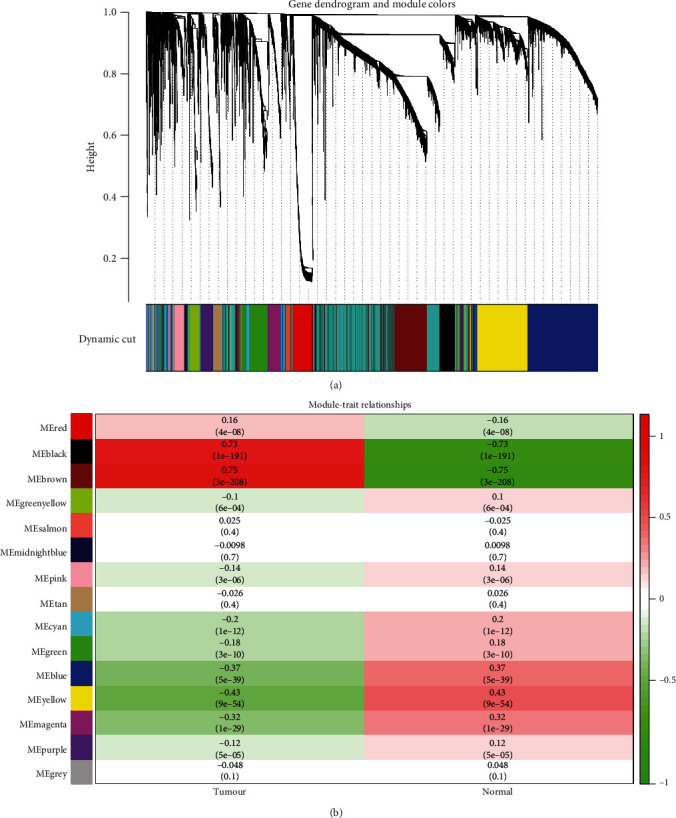
Network analysis of gene expression. The dendrogram was produced by average linkage hierarchical clustering of 2134 differentially expressed RNAs based on weighted gene coexpression network analysis. The different colors represent different clustering modules.

To elucidate functional clusters of DERNAs in BC patients, the gene coexpression network of 2134 DERNAs was analyzed by WGCNA. We specified some key settings (beta = 6, constant height cut‐off = 15) to improve accuracy. Fifteen color-coded coexpression modules were identified (Figure 2(a)). The relation between each module and the clinical status of the corresponding BC sample were calculated and plot to measure the correlation of DERNAs in the network. The results are shown in Figure 2(b). Brown modules were selected owing to their high positive correlation with cancerous specimens. The brown module with the highest module-trait relationship score was deemed to be the core module. The core module with protein-protein interaction (PPI) and functional annotation was continued to be analyzed.

**Figure 3:**
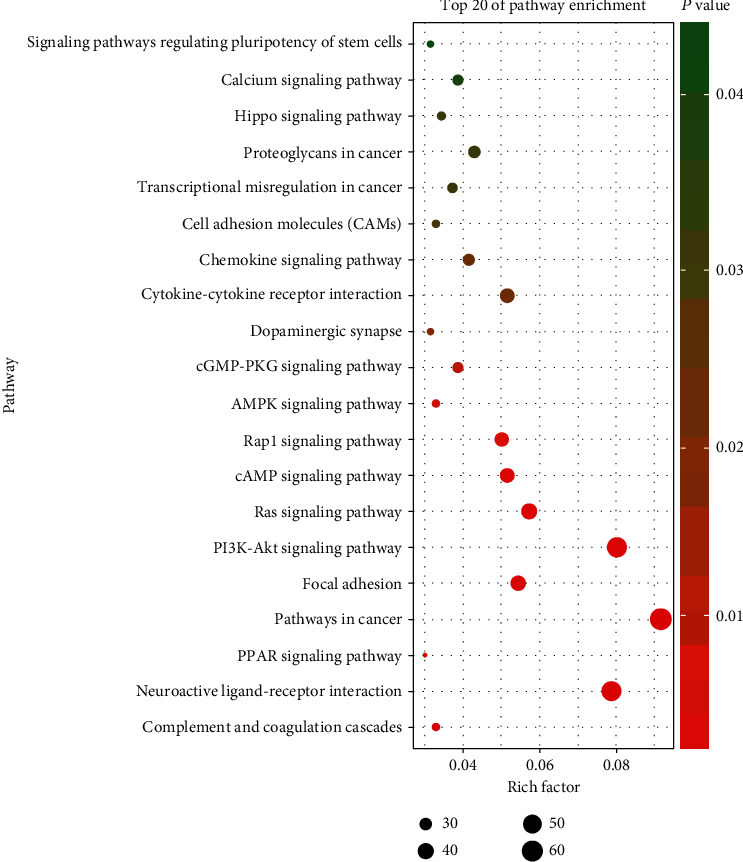
Kyoto Encyclopedia of Genes and Genomes enrichment analysis of brown modules with the significantly enriched biology terms.

The brown (core) module contained 1877 genes with 1464 nodes and 10091 edges. Some genes were core to PPI including GNG2, GNG3, GNG13, GNG11, and SAA1. Gene Ontology includes three components, namely, biological vprocess (BP), molecular function, and cellular component [14]. We selected 15 of the most important enrichment results of the three categories for analysis. The main characteristics of enrichment in the biological process include cell adhesion, the cell surface receptor signaling pathway, positive regulation of cell proliferation, angiogenesis, and the oxidation-reduction process, which are closely related to the growth and proliferation of tumor cells. The process of enrichment in molecular function primarily includes transcriptional activator activity, RNA polymerase II core promoter proximal region sequence-specific binding, and calcium ion binding. The most important characteristics related to the cellular component are the integral component of membrane and plasma membrane, cell surface, and extracellular region. The 1877 genes were mainly enriched in 10 signaling pathways with *P* < 0.01 (Figure 3), including many cancer-related signaling pathways, for example, proteoglycans, PI3K-Akt, Ras, and transcriptional misregulation.

### 3.3. Establishment and Analysis of ceRNA Network in BC

**Figure 4:**
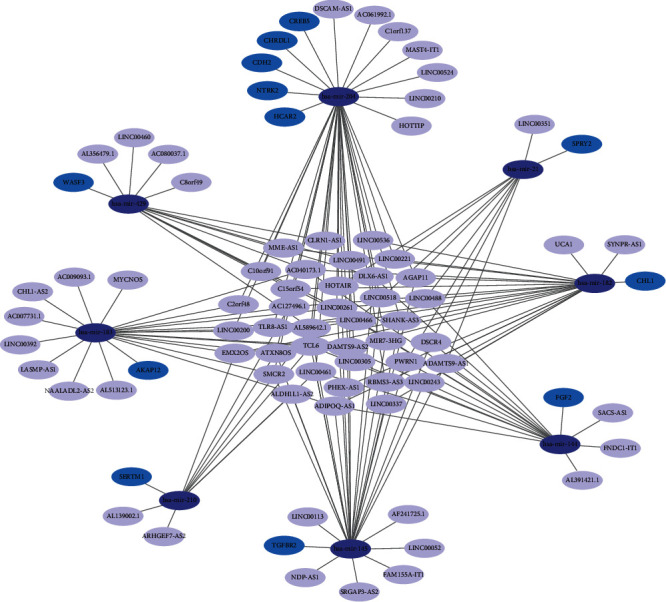
The lncRNA-mRNA-miRNA ceRNA network.

Based on potential links between miRNA-lncRNA and miRNA-mRNA, the correlations between lncRNAs and miRNAs were predicted by using miRanda. miRWalk, TargetScan, and miRDB were employed to find target mRNAs of miRNAs. The results showed that the ceRNA network consists of 72 lncRNAs, 8 miRNAs, and 12 mRNAs (Figure 4). It is reported that majority of the mRNAs in the network are BC-related genes, for instance, NTRK2, CDH2, TGFBR2, and SPRY2. In the network, 11 target mRNAs (NTRK2, SERTM1, TGFBR2, SPRY2, CREB5, FGF2, WASF3, CHRDL1, HCAR2, CHL1, and AKAP12) are downregulated, while one target mRNA (CDH2) is upregulated. Each miRNA had a corresponding mRNA, and Hsa-mir-204 had the most target mRNAs.

### 3.4. Verify the Survivability of lncRNAs, MicroRNAs, and mRNAs of the ceRNA

**Figure 5:**
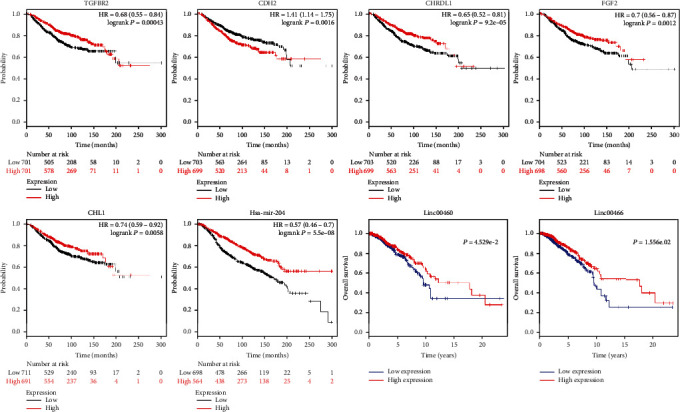
Kaplan-Meier survival curves of 5 mRNAs, 1 miRNA, and 2 lncRNAs associated with the overall survival in breast cancer.

The overall survival rates of mRNAs, lncRNAs, and miRNAs corresponding to the ceRNA network were studied by Kaplan-Meier (*P* < 0.05). Two lncRNAs (LINC00466, LINC00460), 1 miRNA (Hsa-mir-204), and 5 mRNAs (TGFBR2, CDH2, CHRDL1, FGF2, and CHL1) met the survival significance criterion for BC (Figure 5). Based on the ceRNA network, we identified that the LINC00466-Hsa-mir-204-TGFBR2, LINC00466-Hsa-mir-204-CDH2, and LINC00466-Hsa-mir-204-CHRDL1 axes consisted of 10 RNAs related to the prognosis of BC.

**Figure 6:**
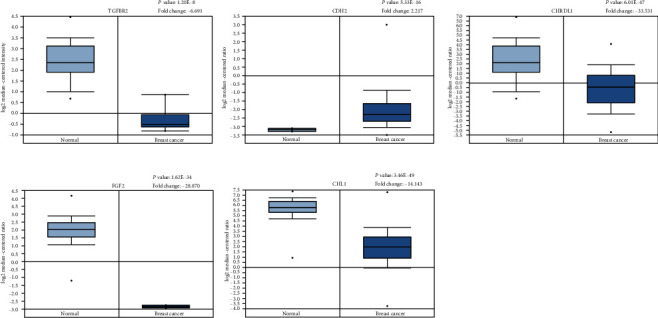
Validation of the expression of TGFBR2, CDH2, CHRDL1, FGF2, and CHL1 was detected in the Oncomine database.

Furthermore, Oncomine was used to determine the transcription levels of mRNA in BC between tumors and normal tissues. The results indicated that 5 mRNAs (CDH2, CHRDL1, TGFBR2, FGF2, and CHL1) were abnormally expressed (Figure 6). CDH2 showed high expression in BC tissues, while CHRDL1, TGFBR2, FGF2, and CHL1 showed low expression. The results are consistent with the Kaplan-Meier analysis.

## 4. Discussion

As one of the most popular female cancers worldwide and the leading cause of cancer-related mortality, it is of great significance to research the molecular interaction of BC [15]. The BC-associated lncRNA-miRNA-mRNA ceRNA network bioinformatics research was performed to explore molecular pathogenesis of BC. We used WGCNA to identify gene modules highly associated with BC and extracted the key genes in the modules. We identified the brown module with the highest association with BC, which contained 1877 DERNAs. We performed functional annotation to define the possible functions of the DERNAs. The results showed that the functions of the 1877 DERNAs were closely related to the growth and proliferation of tumor cells, such as cell adhesion, the cell surface receptor signaling pathway, and positive regulation of cell proliferation. The KEGG results included many cancer-related signaling pathways, for example, proteoglycans, PI3K-Akt, and transcriptional misregulation. This provided the genes for our construction of ceRNAs. Furthermore, based on potential links between miRNA-lncRNA and miRNA-mRNA, the ceRNAs were established. In total, 72 lncRNAs, 8 miRNAs, and 12 mRNAs were obtained. After analysis of total survival and verification, 2 lncRNAs (LINC00460, LINC00466), 1 miRNA (Hsa-mir-204), and 5 mRNAs (CDH2, CHRDL1, TGFBR2, FGF2, and CHL1) were found to be related to the BC patient survival rates.

The ceRNA hypothesis was considered to be a new regulatory mechanism, between noncoding RNA and coding RNA [16]. The lncRNA regulates gene expression by interacting with miRNA binding sites [17]. It may regulate gene expression by transcriptional, posttranscriptional, and epigenetic levels. We explore the validity of DElncRNAs in the ceRNA. Two lncRNAs (LINC00460, LINC00466) were related to the prognosis of BC. LINC00460 has previously been proven to be correlated with the occurrence, development, hyperplasia, and metaptosis of tumors. Feng et al. indicated that LINC00460 was overexpressed in most tumor tissues and esophageal cancer cell lines [18]. Experiments found that LINC00460 depletion suppressed cell growth in esophageal squamous cell carcinoma through regulating cell proliferation and the cell cycle and accelerated cell apoptosis in esophageal squamous cell carcinoma. LINC00460 expression was positively correlated with esophageal squamous cell carcinoma TNM stage and lymph node metastasis and predicted poor prognosis. This is consistent with the results of our survival analysis. LINC00460 was higher in BC patients than in normal samples. Furthermore, a great deal of research has identified that LINC00460 plays an important role in gastric cancer, papillary thyroid carcinoma, colorectal cancer, and other malignant tumors [19, 20]. It can be applied in clinic as a new diagnostic and prognostic indicator. Limited research has been done on LINC0466, but coincidentally, Gao et al.'s research established that the ceRNA correlation with prognosis of invasive breast cancer included the LINC0466-Hsa-mir-204-NTRK2 axis [21]. She indicated that LINC00466, Hsa-mir-204, and ntrk2 took part in the molecular process of invasive breast cancer. Moreover, LINC00466 and Hsa-mir-204 are also predicted to be highly associated with BC in our study.

The miRNAs play a significant part in the regulation of the posttranscriptional gene, which can reveal the process of gene information expression. miRNAs are involved in multiple biological processes including cell proliferation, differentiation, apoptosis, migration, and development [22]. Recent researches have proved that miRNAs are dysregulated in many kinds of human tumors and act as oncogenes or tumor suppressors. Further, miRNAs play an important role in BC as they are confirmed to take part in cancer development, especially in preventing apoptosis and promoting uncontrolled cell division [23].

In this research, we have detected that Hsa-mir-204 was concerned with the prognosis of BC. The miR-204 was first deemed as an antioncogene and is confirmed to be downregulated in lung cancer, glioma, and gastric cancer [24, 25]. Using experimental analysis, Shen et al. showed that miR-204 can target FOXA1 directly to adjust the biological progress in BC cells [26]. Chen et al. used PCR to measure the expression level of serum miR-204 in patients with gastric cancer [27]. The results indicated that the level of miR-204 was obviously lower than that in the normal control group. Serum miR-204 was related to lymph node metastasis, tumor differentiation, and TNM stage. Furthermore, Hsa-mir-204 can contribute to evaluating the state of illness in patients with hepatocellular carcinoma, thyroid cancer, and other cancers [28, 29]. Fan et al. showed that miR-204 was downregulated in all breast cancer cell lines [30]. miR-204 inhibits the overexpression of these cells in the MCF7 breast cancer cell line by inducing apoptosis and G2/M cell cycle arrest. In addition, overexpression of miR-204 inhibited the migration and invasion of MCF7 breast cancer cells. He concluded that miR-204 may be an important therapeutic target for BC. Therefore, the molecular pathogenesis of Hsa-mir-204 in BC needs further study.

The expression of genes was regulated by miRNAs primarily via targeted mRNA degradation. Based on the module results obtained by WGCNA, numerous hub genes in the brown module, for instance, CAVIN2, VEGFD, CHRDL1, and SPRY2, as well as nonhub genes TGFBR2, CDH2, CHRDL1, FGF2, and CHL1, were considered important target proteins. TGFBR2, a direct target of Hsa-mir-204, has recently emerged as a target for cancer therapy [31]. Wei et al. applied PCR, western blot assay, and immune histochemical staining for 65 primary tumor samples and breast tissue samples to ascertain the expression levels of TGFBR2 [32]. The outcomes found that the expression of TGFBR2 mRNA and protein was obviously reduced in BC tissue compared with nontumor tissue. The low TGFBR2 expression was closely related to adverse pathological parameters and poor prognosis in BC. Our results also showed that TGFBR2 is underexpressed in BC. N-cadherin (CDH2) is a kind of cadherin. The changes in its structure or function may hinder the development and maintenance of normal mammary gland cells, leading to the occurrence of breast cancer [33]. Ailan et al. have reported that AP-2*γ* binding sites on genomic DNA of human BC cells were isolated by chromatin immunoprecipitation and identified CDH2 as the target genes in a carcinogenesis group [34]. Thus, CDH2 can be used as a new therapeutic target for future anti-BC.

FGF2 (fibroblast growth factor 2) was reported to be overexpressed in many types of human cancer, for example, gastric cancer, lung cancer, and BC [35, 36]. Giulianelli et al. found that FGF2 was activated by hormone-independent Er*α* and PR through gene sequence. They interact with Myc enhancer and proximal promoter to induce breast cancer cell proliferation. Myc inhibitor, antiestrogen, or antiprogesterone can reverse the effect of FGF2 induction [37]. Ana et al. studied the role of FGF2 subtype in the progression of BC by evaluating the expression and location of FGF2 in 81 cases of BC [38]. They found that FGF2 was expressed in 55.6% of breast cancer cells. There was no significant difference between the expression of nuclear FGF2 and Ki67 proliferation index, tumor stage, and tumor grade. In the low-level tumor samples, the middle- to high-level FGF2 was related to cancer with a low progesterone receptor A isoform than B isoform ratio. Targeting intracellular FGF2 may help to overcome BC progression. The above study shows the significance of FGF2 in BC.

Cyr-Depauw et al. established through live-cell migration assays that high chordin-like 1 (CHRDL1) expression was related to better clinical prognosis in BC patients [39]. Moreover, CHRDL1 antagonizes the function of BMP4 by binding to it and preventing its interaction with receptors, resulting in tumor-suppressing effects in patients with BC. CHL1 deficiency promotes tumor formation in vivo, and CHL1 is downregulated in human BC. Furthermore, knockdown of CHL1 expression causes increased proliferation and invasion in breast cancer cells. Therefore, CHL1 is closely related to BC. The specific mechanism needs further discussion.

According to the constructed ceRNA, we compared 10 prognostic markers associated with BC. We acquired three significative lncRNA-miRNA-mRNA accommodative axes, LINC00466-Hsa-mir-204-TGFBR2, LINC00466-Hsa-mir-204-CDH2, and LINC00466-Hsa-mir-204-CHRDL1. This indicated that LINC00466, Hsa-mir-204, TGFBR2, CDH2, and CHRDL1 participated in the pathogenesis of BC. However, previous reports showed that Hsa-mir-204, TGFBR2, CDH2, and CHRDL1 go hand in hand with the molecular mechanism of BC. Therefore, LINC00466 is an lncRNA that can be used to predict BC and may be regarded as the target for BC.

## 5. Conclusion

In the research, we structured a BC-associated ceRNA network, consisting of 72 lncRNAs, 8 miRNAs, and 12 mRNAs. Based on the analysis and validation of the overall survival rate, we obtained eight prognostic biomarkers associated with BC, including 2 lncRNAs (LINC00466, LINC00460), 1 miRNAs (Hsa-mir-204), and 5 mRNAs (TGFBR2, CDH2, CHRDL1, FGF2, and CHL1). Through comparison and integration, we obtained three axes that relate to the treatment and prognosis of BC, namely, LINC00466-Hsa-mir-204-TGFBR2, LINC00466-Hsa-mir-204-CDH2, and LINC00466-Hsa-mir-204-CHRDL1. This further confirmed the effect of lncRNA, miRNA, and mRNA interaction on the pathogenesis in BC. These findings highlight the abnormal lncRNA-miRNA-mRNA ceRNA related to the pathogenesis of BC, which might be used for the purpose of potential diagnostic biomarkers and therapeutic targets for BC.

## Data Availability

TCGA database website is https://www.cancer.gov/about-nci/organization/ccg/research/structural-genomics/tcga.
